# Helminth Allergens, Parasite-Specific IgE, and Its Protective Role in Human Immunity

**DOI:** 10.3389/fimmu.2014.00061

**Published:** 2014-02-14

**Authors:** Colin Matthew Fitzsimmons, Franco Harald Falcone, David William Dunne

**Affiliations:** ^1^Department of Pathology, University of Cambridge, Cambridge, UK; ^2^School of Pharmacy, University of Nottingham, Nottingham, UK

**Keywords:** helminth, allergen, *Schistosoma mansoni*, protective role, IgE

## Abstract

The Th2 immune response, culminating in eosinophilia and IgE production, is not only characteristic of allergy but also of infection by parasitic worms (helminths). Anti-parasite IgE has been associated with immunity against a range of helminth infections and many believe that IgE and its receptors evolved to help counter metazoan parasites. Allergens (IgE-antigens) are present in only a small minority of protein families and known IgE targets in helminths belong to these same families (e.g., EF-hand proteins, tropomyosin, and PR-1 proteins). During some helminth infection, especially with the well adapted hookworm, the Th2 response is moderated by parasite-expressed molecules. This has been associated with reduced allergy in helminth endemic areas and worm infection or products have been proposed as treatments for allergic conditions. However, some infections (especially *Ascaris*) are associated with increased allergy and this has been linked to cross-reactivity between worm proteins (e.g., tropomyosins) and highly similar molecules in dust-mites and insects. The overlap between allergy and helminth infection is best illustrated in *Anisakis simplex*, a nematode that when consumed in under-cooked fish can be both an infective helminth and a food allergen. Nearly 20 molecular allergens have been isolated from this species, including tropomyosin (Ani s 3) and the EF-hand protein, Ani s troponin. In this review, we highlight aspects of the biology and biochemistry of helminths that may have influenced the evolution of the IgE response. We compare dominant IgE-antigens in worms with clinically important environmental allergens and suggest that arrays of such molecules will provide important information on anti-worm immunity as well as allergy.

## The IgE Response is a Physiological Immune Response to Helminth Infection

The parallels between allergy and the immune response to parasitic worms (helminths) have been noted for some time. Unlike most other inflammatory/infectious conditions, allergy, and helminths induce strongly Th2-skewed responses associated with cytokines such as IL-4, IL-5, and IL-13, with mastocytosis, eosinophilia, and antibody class-switching to produce IgE [reviewed in Ref. (1)]. This normally rare, tightly controlled antibody isotype is greatly elevated in helminth infection. It is widely accepted that IgE, its receptors and distinctive cellular responses did not evolve to target harmless molecules occurring in plant pollen, dust-mites, or animal dander. Instead many believe that the IgE axis evolved to counter metazoan parasites (worms and parasitic arthropods) which are too large to be phagocytosed, and that allergy is a misdirected anti-parasite response in hypersensitive people (2). The symptoms of allergic responses; lachrymation, rhinitis, coughing, increased mucus production, and itching in response to histamine release are all responses likely to dislodge, trap, or flush out large parasites from skin or mucosa, e.g., by scratching.

There are however critical differences between the two conditions. Allergy occurs in people with atopy; defined as “a genetic predisposition toward the development of immediate hypersensitivity reactions against common environmental antigens” (3). It is a polygenic disorder linked to polymorphisms in genes of cytokine, cytokine receptors, and transcription factors associated with Th2 immune responses and with the expression of IgE and its receptors (4–7). In contrast, the elevated Th2 cytokines, IgE and eosinophilia during helminth infection are normal physiological responses to these pathogens. Furthermore, helminths actively moderate the inflammatory Th2 response of the host, inducing regulatory T and B cells, alternatively activated macrophages and production of immunoregulatory cytokines, such as IL-10 and TGFß, as well as IgG4 antibodies that counteract IgE [reviewed in Ref. (8)].

Recently Medzhitov and colleagues (9) re-appraised the toxin hypothesis of allergy (10), proposing that the IgE-mediated hypersensitivity response evolved to counter venoms and other noxious substances rather than macro-parasites. They argued that (1) immediate hypersensitivity is very rapid and worms are slow, (2) IgE is not required for worm immunity in mice, and (3) allergens do not have any obvious relationship with worms. Instead they proposed that it is toxins and venoms that need to be rapidly neutralized and that unpleasant allergic symptoms provoke toxin-avoidance behavior. This “toxin hypothesis” of allergy can in fact be traced back to the original discovery of anaphylaxis by Portier and Richet (11) [reviewed in (12)]. However, we would argue that (1) defense against invading helminth larvae also requires very fast responses – as elegantly demonstrated in the film of *Schistosoma mansoni* cercariae penetrating and moving rapidly through skin tissue (13). Most recently, work by Obata-Ninomiya in Karasuyama’s group (14) has demonstrated the importance of IgE (via ablation of the high affinity receptor) on basophils (but not mast cells) in trapping invading *Nippostrongylus brasiliensis* larvae in the skin of mice.

While it can be shown that IgE is not strictly necessary for anti-worm immunity in mice [argument (2) above], it needs to be stressed that there are other immunity mechanisms operating as well; IgE is a late mammalian additional mechanism to the Th2-mediated mechanisms of lower vertebrates (which are nonetheless still present in mammals), thus IgE-immunity is not the only mechanism of immunity against metazoan parasites available to mammals. This is exemplified by the occurrence of Th2-like immune responses to helminth infection in avian hosts in the absence of IgE (15). Finally [argument (3) above], we propose here that nearly all known allergens have equivalents (of widely varying structure) in metazoan parasites.

Most of the evidence relating IgE to anti-helminth immunity comes from epidemiological data. In a number of studies on human schistosomiasis, levels of anti-parasite IgE have been correlated with resistance to infection (16–22). Anti-parasite IgE responses have also been associated with immunity in human infections with hookworms (23, 24), *Trichuris* (25), and *Ascaris* (26, 27). Human experimental infection with a single, low dose of *Necator americanus* larvae in the context of helminth immunotherapy trials has shown that peripheral blood basophils become sensitized to parasitic allergens within 6 weeks of exposure, and remain fully responsive to stimulation with hookworm allergens years after this single infection (28). Thus, it appears that helminths are indeed powerful inducers of an IgE response, but how does this response relate to allergy?

## Effects of Helminths on Allergy

Paradoxically, the global increase in allergy especially in urban areas (29) has led researchers to propose a modified hygiene hypothesis in which the decline in helminth infections is associated with an increase in allergic diseases (30). A number of studies show that communities with helminth infections have reduced rates of allergy (31–33) and the evidence that people with hookworm have less asthma (34–36) has inspired researchers to use experimental infections on asthma patients (37). It is proposed that the active suppression of Th2 responses by helminths has a bystander effect on concurrent allergic responses [reviewed in Ref. (8)]. In a study on Gabonese children, van den Biggelaar et al. (31) showed that the increased IL-10 levels induced by schistosome infection were negatively correlated with dust-mite sensitivity. The other side of these phenomena is that anti-helminth treatment programs risk increased rates of allergic disease and this has already been demonstrated in a number of intervention studies (38–40).

Under some circumstances helminth infection can actually increase prevalence of atopic disease and asthma (41, 42). A meta-analysis of 30 clinical studies on intestinal nematodes, concluded that while hookworm reduced the incidence of asthma, *Ascaris lumbricoides* increased the risk (34). It is likely that cross-reactivity between *Ascaris* and environmental allergens is involved.

The concept of cross-reactivity between helminth and environmental allergens is central to this review. We suggest that most if not all environmental allergens can be related to helminth counterparts and that the IgE response against these allergens is associated with host protection.

## Are all Allergens Proteins with Homologs in Metazoan Parasites?

Work in the allergy field has shown that very few protein families contain allergens (43) and importantly, the molecules targeted by IgE in helminths appear to be in these known allergen families (see Tables 1 and 2). Certain domains are highly represented in the list of known molecular allergens with the 10 most common allergen families containing approximately 40% of all know allergens. In the following section, we review the relationship between known helminth allergens and the structural allergen classification in the allergen database AllFam (http://www.meduniwien.ac.at/allergens/allfam).

**Table 1:** **Summary of helminthic allergens**.

Helminth allergen	Common name	Gene ontology (biological process)	Related common allergen	Conserved domains	UniProt accession number	AllFam	Reference
***Anisakis simplex* (HERRING WORM)**							
Ani s 1	Serine protease inhibitor (Kunitz type)	Serine protease inhibitor	Aprotinin	BPTI/Kunitz family of serine protease inhibitor cd00109	L7V3Q3	AF003	Moneo et al. (44)
Ani s 2	Paramyosin	Motor activity	Panallergen	Myosin tail PF01576	L7V1I9	AF100	Pérez-Pérez et al. (45)
Ani s 3	Tropomyosin	Troponin T binding	Panallergen	Tropomyosin PF00261	Q9NAS5	AF054	Asturias et al. (46)
Ani s 4	Cystatin	Cysteine type endoprotein type inhibitor	Minor cat allergen (Fel d3)	Cystatin-like domain cd00042	Q14QT4	AF005	Moneo et al. (47)
Ani s 5	SXP/RAL-2	Unknown	Unknown	PF02520/DUF148	A1IKL2	AF137	Kobayashi et al. (48)
Ani s 6	Trypsin inhibitor like cysteine rich domain	Trypsin inhibitor like cysteine rich domain	Minor latex allergen (Hev b SPI)	Trypsin inhibitor like cysteine rich domain PF01826	A1IKL3	n/a	Kobayashi et al. (48)
Ani s 7	n/a	Unknown	Unknown	None	A9XBJ8	n/a	Rodríguez et al. (49)
Ani s 8	SXP/RAL-2	Unknown	Unknown	DUF148 PF02520	A7M6S9	AF137	Kobayashi et al. (48)
Ani s 9	SXP/RAL-2	Unknown	Unknown (As14 ascaris allergen)	DUF148 PF02520	B2XCP1	AF137	Rodriguez-Perez et al. (50)
Ani s 10	Unknown	Unknown	Unknown	Unknown	D2K835	n/a	Caballero et al. (51)
Ani s 11	Unknown	Unknown	Unknown	Unknown	E9RFF3	n/a	Kobayashi et al. (52)
Ani s 12	Unknown	Unknown	Unknown	Unknown	L7V0K0	n/a	Kobayashi et al. (52)
Ani s CCOS3	Cytochrome *c* oxidase subunit 3	Aerobic electron transport chain	Bermuda grass pollen allergen 46 kDa (Cyn d Bd46k)	Cytochrome *c* oxidase subunit III cd01665	Q1 × 6K9	n/a	López and Pardo (53)
Ani s Cyt B	Cytochrome *b*	Aerobic electron transport chain	Unknown	Cytochrome *b* (N-terminus)/b6/petB cd00284	Q1 × 6L0	n/a	López and Pardo (53)
Ani s FBPP	Fructose 1,6-bisphosphatase	Phosphatase activity	Unknown	n/a	n/a	n/a	López and Pardo (53)
Ani s NADHDS4L	NADH dehydrogenase subunit 4L	NADH dehydrogenase	Unknown	ND4L cl10160	Q1 × 6K2	n/a	López and Pardo (53)
Ani s NARaS	Nicotinic acetylcholine receptor alpha-subunit	Unknown (nicotinic acetylcholine receptor)	Unknown	n/a	n/a	n/a	López and Pardo (53)
Ani s PEPB	(Phosphatidyl-ethanolamine-binding Protein)	Unknown (phosphatidyl-ethanolamine-binding)	Unknown	n/a	n/a	n/a	López and Pardo (53)
Ani s Troponin	Troponin C	Calcium ion binding	German cockroach allergen (Bla g 6)	EF-hand Ca^2+^ binding motif PF00036	Q9U3U5	AF007	Arrieta et al. (54)
***Schistosoma mansoni* (BLOOD FLUKE)**							
Sch ma PM	Paramyosin	Motor activity	Panallergen	Myosin tail, PF01576	P06198	AF100	Webster et al. (55)
Sch ma Sm20	CBP, Sm20.8, Sm20	Calcium ion binding	Unknown	EF-hand Ca^2+^ binding motif, PF00036	P91804	n/a	Fitzsimmons et al. (56)
Sch ma Sm21	SmTAL2, Sm21.7	Calcium ion binding	Unknown	EF-hand Ca^2+^ binding motif PF00036	P32070	n/a	Fitzsimmons et al. (56)
Sch ma Sm22	SmTAL1, CBP	Calcium ion binding	Unknown	EF-hand Ca^2+^ binding motif, PF00036	P14202	n/a	Webster et al. (57)
Sch ma Sm31	Sm31, SmCB1, cathepsin B-like cysteine proteinase	Proteolysis, regulation of catalytic activity	Papain	Papain family cysteine protease, PF00112	P25792, Q8MNY2, G4V5C2, Q8MNY1, G4V5C1, G4V5D0	n/a	de Oliveira Fraga et al. (58)
Kappa-5	k-5	Unknown	Unknown	Unknown	AAX83114.1	n/a	Schramm et al. (59)
***Necator americanus* (HOOKWORM)**							
Nec a ASP-2	ASP-2	Unknown	Unknown	SCP-like extracellular protein domain, cd00168	Q7Z1H1	n/a	Zhan et al. (60)
Nec a calreticulin	Calreticulin	Calcium ion binding	Unknown	Calreticulin superfamily, PF00262	O76961	n/a	Pritchard et al. (61)
***Ascaris*** ***suum* (PIG ROUNDWORM) AND *Ascaris lumbricoides* (HUMAN ROUNDWORM)**							
Asc s 1	ABA-1, nematode polyprotein allergens	Fatty acid and retinoid binding	Unknown	n/a	Q06811	n/a	Christie et al. (62)
Asc s3	Tropomyosin	Troponin T binding	Panallergen	Tropomyosin, PF00261	F1L5K1, F1L3V2, F1KVZ5, F1L218	n/a	Acevedo et al. (63)
GSTA	Glutathione *S*-transferase 1	Transferase	Dust-mite allergen, Der p 8	GST_C_Sigma_like, cd03039, PF13417, GST_N_Sigma_like, cd03192, PF02798	P46436	n/a	Acevedo et al. (64)
***Echinococcus granulosus* (DOG TAPEWORM)**							
AgB	Antigen B	n/a	Unknown	n/a	n/a (multigene family)	n/a	Vuitton, (65)
Ag5	Antigen 5	Proteolysis	Unknown	Trypsin-like serine protease, PF00089, cd00190	A2MJI2, I1WXU1	n/a	Khabiri et al. (66)
EA21	Cyclophilin	Protein folding	*Malassezia furfur* allergen, Mal f 6	Cyclophilin_ABH_like, cd01926	P14088	AF038	Ortona et al. (67)
HSP70	Heat shock protein 70	Response to stress	Dust-mite allergen Hsp70	Hsp70 PF00012	Q24789	AF002	Ortona et al. (68)
EF-1 beta/delta	EF-1	Translation elongation factor	Unknown	Elongation factor 1 beta (EF1B) guanine nucleotide exchange domain	Q9U8D5, Q9NGP3, Q0PWC5	n/a	Ortona et al. (69)
***Brugia malayi* (MALAYAN FILARIA)**							
Bru m 3	Tropomyosin	Troponin T binding	Panallergen	Tropomyosin, PF00261	A8NGJ2	n/a	Sereda et al. (70)
Bru m 13	GST, glutathione *S*-transferase	Metabolic process	House dust-mite allergen Der p 8	GST_N family cd03076, GST C-terminal domain family cd03210	A8PTL9, O02636	n/a	Rathaur et al. (71)
Bru m Bm33	Aspartic protease inhibitor, Bm33	Unknown	Unknown	Ascaris pepsin inhibitor-3 (API3) cl11634	A8Q4E4	n/a	Krushna et al. (72)

*This table was compiled mainly from data extracted from the Allergome database (73) in combination with published literature. Conserved domain annotation is from conserved domain database (CDD) (74) and Pfam (PF; DUF, domain of unknown function) (75). AllFam numbers (AF) are from the database of allergen families AllFam (43). As can be seen from this table, not all helminth allergens currently have related common (non-helminthic) allergens. For example, there are currently no known common environmental allergens structurally related to the nematode polyprotein allergens*.

**Table 2:** **Examples of known allergens, compiled from AllFam (43) and published literature, illustrating that nearly all families of allergens in animals, plants, or fungi have corresponding allergens in helminths**.

Structural motif (AllFam Acc.)	Parasite allergens	Plant allergens	Animal allergens (non-helminth)	Fungal allergens
Tropomyosin (AF054)	Ani s 3, Asc s 3, Bru m 3, Onc v 3, Onc o 3	–	Bla g 7, Blo t 10	–
Paramyosin (AF100)	Ani s 2, Sch j PM, Sch ma PM	–	Blo t 11, Der f 11, Der p 11	–
CRISP/PR-1/venom group 5 (AF044)	Na ASP-2, SmVAL4 (?)	Art v 2, Cyn d 24	Dol a 5, Pol a 5, Pol d 5, Ves g 5, Vesp m 5	–
EF-hand (AF007)	Sm TAL1, Ani s Troponin	Bet v 3, Bet v 4, Art v 5, Par j 4, Phl p 7	Cyp c 1, Gad m 1, Sal s 1, Thu a 1	–
Glutathione *S*-transferase (AF010)	Wb GST^a^, Bru m 13, Onc v 13, Asc l 13, Asc s 13	Tri a GST	Bla g 5, Der p 8, Blo t 8	Asp f GST, Pen c 24
Nematode Polyproteins (n/a)	ABA-1 (Asc s 1) Gp 15/400	–	–	–
Cyclophilin (AF038)	EA21 (*E. granulosus*)	Bet v 7, Cat r 1	–	Asp f 11, Mala s 6
Hsp70 (AF002)	Hsp70 (*E. granulosus*)	Cor a 10	Der f HSP70	Alt a 3, Cla h HSP70
Calreticulin (AF055)	Na Calreticulin	–	–	Pen ch 31
Kunitz Trypsin inhibitor (AF003)	Ani s 1	Gly m TI, Sola t2, Sola t3, Sola t4	Bos d 3, Bos d TI (aprotinin)	–

*As can be seen from this table, there currently appear to be no known non-helminth allergens corresponding to the nematode polyprotein family, although similar biological lipid binding functions are found, e.g., in the lipocalin allergen family (AF015)*.

*^a^N-terminal domain similar to C-terminal domain of glutathione *S*-transferase (AF010)*.

For example, the muscle protein tropomyosin (AllFam code AF054) is an important IgE target in a number of nematode infections; *Onchocerca volvulus* (76, 77); *Ascaris lumbricoides* (78); *Anisakis simplex* [Ani s 3, (46)]; and tropomyosin from the blood fluke *Schistosoma mansoni* is also a human IgE antigen (Fitzsimmons, unpublished data). Tropomyosin is highly conserved across many invertebrates and is responsible for much of the IgE cross-reactivity between *Ascaris* and dust-mites (63). Cockroach tropomyosin is a major allergen (Bla g 7) that also shows strong IgE cross-reactivity with the highly similar *Ascaris* molecule (78). Santiago and co-authors (77) showed that tropomyosin from filarial nematodes is recognized by IgE against dust-mite tropomyosin (Der p 10), which can be absorbed completely using the nematode molecule. More importantly, they showed that the IgE response to Der p 10 was stronger in filarial-infected than in uninfected individuals.

Paramyosin is another allergen family (AF100) from invertebrate muscle targeted in IgE responses against *Schistosoma japonicum* (20), *Ascaris lumbricoides* (79), *Anisakis simplex* [Ani s 2 (45, 80)], and *Onchocerca volvulus* (81). There is evidence that *Ascaris* paramyosin shows IgE cross-reactivity with the tropical dust-mite paramyosin and allergen Blo t 11 (79). Cross-reactivity between helminths and environmental allergens has clear implications. Not only may some helminth infections increase sensitivity to mites and insects, but also high degrees of homology between parasite and allergic orthologs could lead to false diagnosis. Human helminth infections are not restricted to tropical regions (82). Ani s 2 and Ani s 3 are thought to be responsible for much of the cross-reactivity between *Anisakis* and other invertebrate species (83).

The helminth venom-allergen-like (VAL) proteins are another family targeted by IgE. Hookworms secrete a VAL-like molecule, called *Ancylostoma* Secreted Protein-2 (ASP-2), which was shown to be a potent IgE antigen in human studies in China and Brazil (24, 84). An IgE response to this molecule has been correlated with immunity (24). ASP-2 belongs to the Pathogen related-1 (PR-1) allergen family (AF044) characterized by the presence of the SCP/TAPS domain (Pfam, PF00188). The family contains group 3 and 5 insect venom allergens and VAL molecules from filarial nematodes, *Onchocerca volvulus* (85), and *Brugia malayi* (86), as well as trematodes *S. mansoni* (87) and *S. japonicum* (88). Furthermore, the presence of VAL molecules is also predicted in tapeworms (89). One of the *S. mansoni* homologs (SmVAL4) has been recently shown to be an IgE antigen in mice (90), but requires confirmation in the natural human host.

**Figure 1:**
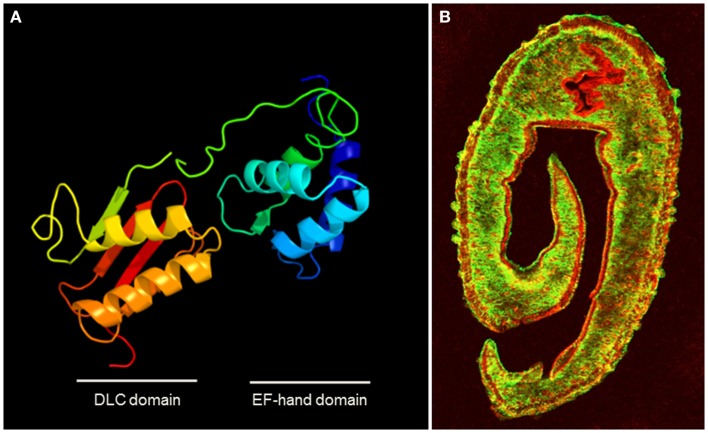
**(A)** Homology modeling of the structure of the dominant SmTAL1 allergen in *S. mansoni* generated using protein homology/analogy recognition engine 2 (PHYRE2) (132), showing the two helix-loop-helix Ca^2+^-binding motifs within the EF-hand domain. **(B)** Transverse section of male *S. mansoni* worm stained for the surface protein SmCD59 (green) and under that in the tegument layer, the EF-hand protein SmTAL1 (red) (courtesy of Prof. Alan Wilson University of York). The walls of the gut also stain for SmTAL1. The location illustrates how this sub-surface allergen in inaccessible to host IgE, unless the tegument layer is damaged, but its physiological function and role in host protection remain to be elucidated.

The tegumental allergen-like (TAL) proteins are some of the most dominant IgE-antigens in *S. mansoni* and an IgE response to some members of the TAL family has been associated with resistance to re-infection with the parasite (18, 19, 22). These molecules are EF-hand proteins (see Figure 1A), one of the biggest groups of molecular allergens (AF007). Other known allergenic helminth EF-hand proteins include *Anisakis simplex* troponin C (54) and the *Fasciola* calcium-binding protein, FgCaBP (91).

The glutathione *S*-transferase (GST) is another source of IgE cross-reactivity. GST of nematode species is targeted by IgE during infection (92). This enzyme is homologous with other members of the GST allergen family (AllFam, AF010) including major allergens in dust-mite (Der p 8) and cockroach (Bla g 5) as well as IgE-antigens in grass and fungi. GST from the filarial nematode *Wuchereria bancrofti* binds IgE against Bla g 5 (77).

Probably, the most potent helminth allergens are the nematode-polyprotein-antigens (NPA). These are large multimeric proteins that are cleaved into smaller fatty acid binding subunits (93) with functional but not structural similarity to the lipocalin allergens (AF015). The best characterized example is the ABA-1 protein from *Ascaris* species. Highly abundant in the body fluid of the adult worm, it provokes a strong IgE response in many infected individuals (93) and this has been associated with resistance to infection (27). The filarial nematode NPA termed gp15/400 has also been shown to be an IgE antigen (94). Interestingly, the non-NPA lipocalin-like fatty acid binding protein from filarial nematodes, BmA1.1, is an IgE antigen which can induce wheal and flare response in sensitized dogs (95).

While some of the Top 10 allergen families (tropomyosins, EF-hand proteins, PR-1, and lipocalins) have members in helminth species that are known to be targeted by IgE during infection, other common allergen families (profilin, trypsin-like serine proteases, and lipocalin) have been identified in helminths, but their IgE binding has not yet been tested (96). Furthermore, the plant prolamins (AF050) and expansins (AF093 and AF094) are Top 10 allergen families that do not contain helminth equivalents. However, this assessment is made on sequence alignment and it is possible that these plant proteins share conformational motifs formed by non-homologous sequences (mimetopes) in un-related proteins from metazoan parasites. There is some evidence for this in that the plant expansin Php p 1 has no sequence homology with the mite allergen Der p 2, but Phl p 1, and Der p 2 have domains that share function (carbohydrate-binding) and close 3D conformational homology (97). While dust-mites are not metazoan parasites, they have close relatives that are (e.g., the scabies mite, *Sarcoptes scabiei*). Interestingly, the IgE response to *Sarcoptes scabiei* is thought to be involved in protection against repeat infestation (98).

## What Makes an Antigen an Allergen?

Perhaps the greatest unanswered question in allergy is why only a small minority of antigens has allergenic properties. As stated previously, most proteins are not allergens. Thus, there are currently almost 15,000 protein domain families in the Pfam database (http://pfam.sanger.ac.uk/) of which only 255 have been identified in allergens (http://www.meduniwien.ac.at/allergens/allfam). The debate about which functional and molecular properties make a protein an allergen has continued for some time (99–101). Some functional properties give environmental and food proteins, a greater chance of sensitizing susceptible individuals. For example, high thermal stability allows allergens to persist in the environment or survive cooking and digestion. This is well illustrated by the example of plant chitinases, which are members of the pathogenesis-related family of proteins 4 (PR-4). Plant *chitinases* (AF041) have been described as panallergens in latex-fruit syndrome and are contained in a multitude of plants, such as Heveine [in latex, (102), kiwi fruit (103), in avocado (Pers a1, (104)] or grapes (105) and are related to dust-mite allergens Der p 15 and Der p 18 (106). Consistently with the hypothesis of thermal stability, despite the ubiquitousness of such PR-4 group proteins across the plant kingdom, allergenicity is only reported in foods that are consumed uncooked, as type I chitinases are inactivated by heating (107). While chitinases are also well represented in non-parasitic as well as parasitic helminths, to the best of our knowledge, no helminthic chitinases have yet been reported as allergens. The reasons for this are not understood.

In relation to food allergens and cooking, the special case of *Anisakis simplex* (*A. simplex*) deserves to be mentioned. *Anisakis* is the only currently known case of an organism being both a helminth parasite and a food allergen. The L3 larvae of the marine nematode *A. simplex* infect fish and cephalopods and consequently people that consume under-cooked seafood, however humans are a non-permissive host and the parasites cannot continue their life-cycle in man. Exposure to this helminth through food has been associated with allergic symptoms; asthma, rhinitis, dermatitis, and conjunctivitis (80), and in the case of uncooked fish, epigastric pain, erythema wheals, and pruritus (“gastroallergic anisakiasis”). It is not clear whether initial sensitization requires live parasite infection (anisakiasis) but it has been shown that sensitized patients can respond to heated or frozen *Anisakis* antigens in their food (108) or to small quantities by other exposure routes (109), such as skin contact, inhalation, or during skin prick testing. That the immune system responds to *Anisakis* as an invading helminth and as an allergen suggests that these are two aspects of the same response.

A feature of a relatively small subset of allergens is their proteolytic activity, which may permit penetration of mucosal barriers (110), for example, by cleaving proteins involved in tight junction formation (111). Many helminthic parasites rely on production of proteases during tissue migration, and we have previously argued that such proteases may be a factor underlying the parasites’ intrinsic allergenicity (112).

However, such biological properties are not always present in allergens and the small percentage of protein domains that are targeted by IgE overall, in the absence of common biological activities, suggests they contain structures that are inherently allergenic. These structures vary widely and appear to have little in common overall. Given the probable evolution of the IgE system, we have proposed that proteins have inherent allergenicity because they have structural similarity to dominant antigens in metazoan parasites (96). However, it still remains unclear how such intrinsic structural features selectively enable a subset of antigens to induce, or become the object of, an IgE response.

Another consideration seems necessary. Many of the allergen families described above are also present in humans, but are not the target of an IgE response. Following *in silico* analysis of animal food proteins and their IgE responses, Jenkins and colleagues proposed that proteins with a sequence identity to a human homolog of >62% were rarely allergenic (113). We believe the IgE system evolved to target Th2 responses at large multi-cellular parasites, organisms that are much more closely related to us that bacterial, fungal, or viral pathogens. This means the evolved molecular targets had to be restricted if foreign metazoan antigens were to be targeted without inducing tolerance or risking auto-reactivity, and that non-parasitic proteins are allergenic because of their homology with metazoan parasites.

The hypothesis was examined by Santiago et al. (114). Using a bioinformatic approach, they compared the sequences of 499 allergens against the predicted proteomes of four helminths (including *Schistosoma mansoni*), four bacterial, and three fungal species. Their analysis supported previous work by Emanuelsson and Spangfort (115) finding little homology between bacterial proteins and allergens and the work by Jenkins et al. (113) who showed a drop in allergenicity as homology with human equivalents increased. While they reported that over 200 allergens had homologs in helminths, this was the minority, and indeed those with the greatest homology were the least allergenic. They concluded that allergenicity does not depend on similarity with parasite proteins, but on dissimilarity with human proteins. It should be remembered however, that most IgE epitopes are probably conformational (discontinuous) (116, 117) and would not be identified in such primary sequence comparisons.

## Life-Cycle Expression of Helminth Allergens and the Host Response

Clinically important helminths often have complex life-cycles. Many involve a definitive host (man) and one or more intermediate hosts. The life-cycle expression profile of allergen-like molecules influences the host response. For example, trematodes (flukes) such as schistosomes undergo asexual reproduction in snail species before releasing larvae that infect humans, which then develop into adult worms that produce eggs following sexual reproduction. Some of the schistosome allergen-like TAL proteins are developmentally transcribed (22). SmTAL1 is sequestered inside the adult worms (Figure 1B) and is only exposed on the rare occasions when the adults die (56). Typically, *S. mansoni* worms live for 7–9 years (118). As individuals are usually infected more than once, resulting in asynchronous development and death of the parasite, this resembles seasonal allergen stimulation and infected people in areas endemic for *S. mansoni* have high levels of IgE to SmTAL1. SmTAL2 is expressed in schistosome eggs. In chronic infection hundreds of parasite eggs are trapped and die in the tissue every day. In a process that resembles specific allergen immunotherapy (SIT), the IgE response to allergen-like SmTAL2 appears to be desensitized by the continuous exposure to small doses of the antigen, while the specific IgG4 response becomes pronounced (56). SmTAL6 is only expressed in the snail stage and has no effect on the human response (119).

Adult tapeworms live in the lumen of the gut shedding eggs for excretion. If these eggs are ingested by a secondary host, they hatch and larvae encyst in the soft tissue. The contents of these structures are highly allergenic and can cause anaphylaxis if they burst. People carrying cysts of *Echinococcus granulosus* (echinococcosis) have IgE to parasite antigens AgB, a protease inhibitor, Ag5, a serine protease, and EA21 (65, 67). EA21 is a cyclophilin that shares close homology with allergenic yeast cyclophilin (Mal f 6) and may be cross-reactive with allergenic birch cyclophilin Bet v 7 (65). Infected individuals also produce IgE to the C-terminal region of *E. granulosus* Heat Shock Protein 70 an antigen with close homology to the dust-mite allergen, Der f HSP70 (68).

Hookworm eggs hatch in the soil where the larvae undergo several molts before becoming the infectious L3 form that penetrates the skin of the foot. The larvae then migrate to the lung and are coughed up, swallowed, and hence taken to their niche in the small intestine. It is the skin-penetrating L3 form that expresses and secretes the VAL protein ASP-2 (120). Since an antibody response to the molecule was associated with reduced infection ASP-2 has been tested as a vaccine candidate (24). Unfortunately, clinical trials in a hookworm endemic region of Brazil had to be stopped when vaccinated volunteers with a probable previous history of infection (as judged by the levels of pre-vaccination parasite-specific IgE) developed symptoms of generalized urticaria (84). The relationship between the protective effects of parasite-specific IgE and the hazards of vaccinating a sensitized population with an allergen present a major conundrum which is currently hampering the development of anti-helminthic vaccinations.

These three examples were chosen to illustrate the concept that allergen expression in helminth parasites is not a generalized feature of parasitic worms but a specific property of distinct developmental phases in the human host which is tightly linked to host protective mechanisms. Anti-protein IgE responses and host defense are two sides of the same coin which in our opinion are inseparable from each other.

However, while the link between the presence of parasite-specific IgE and resistance to infection is well supported by epidemiological and experimental evidence, the detailed molecular basis underlying such resistance is less well understood.

Specifically, one of the great unanswered questions is whether the presence of IgE on FcεRI-carrying cells (mainly basophils, mast cells, eosinophils) and subsequent receptor cross-linking by parasitic allergens is needed for host protection. Is the activation of basophils, mast cells, and other IgE-bearing effector cells necessary for protection?

It is well know that activation of mast cells and eosinophils can release proteases and toxic proteins (chymase, tryptase, major basic protein, eosinophil-derived neurotoxin, eosinophils cationic protein, etc.), some of which have been shown to directly kill larval stages of parasites (121).

Similarly, it could be speculated that IgE-dependent activation of basophils, which can result in the release of preformed or *de novo* produced highly toxic polypeptides such as Granzyme B (122) and possibly defensins (Falcone, unpublished data), also may result in parasite killing. While host-derived defensins have been shown to be effective against several unicellular parasites such as *Plasmodium* (123), *Toxoplasma* (124), *Babesia* (125), or *Trypanosoma* (126), their role in anti-helminthic immunity has only recently begun to be explored (127).

## A Look into the Future: “Molecule-Based” Analysis of Anti-Parasite Host Immune Responses?

Traditionally, immunoparasitological research has relied on the use of complex antigenic mixtures such as somatic extracts of larval or adult stages, of eggs or of the tegument, or excretory/secretory materials collected *in vitro*, which all contain a multitude of antigens, allergens, and other un-related components. This can result in a low signal to noise ratio, for example caused by the presence of highly cross-reactive carbohydrate moieties, masking specific interactions at the individual protein level.

Due to the widespread use of complex water-soluble extracts obtained from parasitic materials in the past decades of parasitology research, several questions still remain to be answered. What are the individual molecular targets of the protective IgE response? Are certain patterns of IgE reactivity (rather than against a single determinant) associated with host protection? Do different IgE reactivity patterns correlate with various degrees of resistance to infection or post treatment re-infection?

This is reminiscent of the situation previously encountered in allergy research, which relied on water-soluble extracts which are difficult to standardize for diagnostic purposes (128), and may contain interfering components. Major impulses in the past years have come from introducing component resolved diagnosis (CRD) to the study of human allergy. In CRD, individual recombinant or purified allergens are used for measurement of immunoglobulin responses in allergic individuals (129). This frequently takes advantage of the availability of protein microarrays (130). One of the key advantages of CRD is that it may enable distinction between genuine IgE reactivity and cross-reactive IgE (131).

While the use of CRD in allergy diagnosis is conceptually slightly different (it is used to identify the allergen source when cross-reactive allergen components are present), we suggest that a similar “molecule-based” approach would allow a better understanding of host resistance against helminths at the molecular level and, from a practical point of view, point the way to safer or more effective multi-target anti-helminthic vaccinations.

## Concluding Remarks

If the IgE axis evolved to protect mammals against multi-cellular parasites, studying host responses to these organisms may teach us much about other IgE-mediated phenomena such as allergy. For example, characterizing parasite structures targeted by IgE may identify homologous molecules and potential allergens in novel foods and genetically modified organisms. The relationship between allergy and helminth infection brings costs and benefits. Elucidation of the molecular mechanisms by which some parasites moderate Th2 response in their hosts, may yield improved therapy for allergic conditions. On the other hand, treatment for the same worms in the developing world may inadvertently increase the prevalence of atopic disease. Moreover as a consequence of cross-reactivity between parasite and environmental allergens certain helminths can actually sensitize and aggravate allergy. Parasitic worm infections are a serious health problem in many countries and high resolution molecular techniques developed in the allergy field may help us to understand better the anti-parasite responses that are associated with immunity.

## Conflict of Interest Statement

The authors declare that the research was conducted in the absence of any commercial or financial relationships that could be construed as a potential conflict of interest.
